# FDrisk: development of a validated risk assessment tool for Fabry disease utilizing electronic health record data

**DOI:** 10.1007/s44162-023-00026-7

**Published:** 2024-01-04

**Authors:** Caryn J. Lobel, Dawn A. Laney, Jingjing Yang, David Jacob, Amy Rickheim, Carol Z. Ogg, Diana Clynes, Jessica Dronen

**Affiliations:** 1 Department of Human Genetics, Emory University School of Medicine, Atlanta, GA USA; 2 Department of Human Genetics, Center for Computational and Quantitative Genetics, Emory University School of Medicine, Atlanta, GA USA; 3 ThinkGenetic Foundation, Inc., Sudbury, MA USA; 4 COCo Marketing Group, Atlanta, GA USA; 5 American Association of Kidney Patients, Tampa, FL USA; 6 ThinkGenetic, Inc., Waynesboro, VA USA

**Keywords:** Fabry disease, FDrisk, Risk score, Risk assessment, Scoring tool, Fabry disease diagnosis

## Abstract

**Purpose:**

Fabry disease (FD) is a rare, X-linked, lysosomal storage disease characterized by great variability in clinical presentation and progressive multisystemic organ damage. Lack of awareness of FD and frequent misdiagnoses cause long diagnostic delays. To address the urgent need for earlier diagnosis, we created an online, risk-assessment scoring tool, the FDrisk, for predicting an individual’s risk for FD and prompting diagnostic testing and clinical evaluation.

**Methods:**

Utilizing electronic health records, data were collected retrospectively from randomly selected, deidentified patients with FD treated at the Emory Lysosomal Storage Disease Center. Deidentified, negative controls were randomly selected from the Fabry Disease Diagnostic Testing and Education project database, a program within the American Association of Kidney Patients Center for Patient Education and Research. Diagnosis of FD was documented by evidence of a pathogenic variant in *GLA* and/or an abnormal level of leukocyte α-Gal A. Thirty characteristic clinical features of FD were initially identified and subsequently curated into 16 clinical covariates used as predictors for the risk of FD. An overall prediction model and two sex-specific prediction models were built. Two-hundred and sixty samples (130 cases, 130 controls) were used to train the risk prediction models. One-hundred and ninety-seven independent samples (30 cases, 167 controls) were used for testing model performance. Prediction accuracy was evaluated using a threshold of 0.5 to determine a predicted case vs. control.

**Results:**

The overall risk prediction model demonstrated 80% sensitivity, 83.8% specificity, and positive predictive value of 47.1%. The male model demonstrated 75% sensitivity, 95.8% specificity, and positive predictive value of 75%. The female model demonstrated 83.3% sensitivity, 81.3% specificity, and positive predictive value of 45.5%. Patients with risk scores at or above 50% are categorized as “at risk” for FD and should be sent for diagnostic testing.

**Conclusion:**

We have developed a statistical risk prediction model, the FDrisk, a validated, clinician-friendly, online, risk-assessment scoring tool for predicting an individual’s risk for FD and prompting diagnostic testing and clinical evaluation. As an easily accessible, user-friendly scoring tool, we believe implementing the FDrisk will significantly decrease the time to diagnosis and allow earlier initiation of FD-specific therapy.

## Introduction

Fabry disease (FD) (OMIM no. 301500) is a rare, X-linked, lysosomal storage disease caused by pathogenic variants in the *GLA* gene which result in an absence or severe reduction of the lysosomal enzyme α-galactosidase (α-Gal A, EC 3.2.1.22). This leads to an excess of glycosphingolipids, mainly globotriaosylceramide (Gb3/GL3) and its deacylated form globotriaosylsphingosine (lysoGb3/lysoGL3) [1, 2] which, along with the associated downstream inflammatory mechanisms [3], cause progressive multisystemic organ damage. Of note, despite being an X-linked condition, females are often significantly affected due to a combination of skewed X-chromosome inactivation, clonal expansion, and somatic mosaicism. As such, females experience severity of disease ranging from asymptomatic to severe, resembling that which can be observed in males [4–7].

FD is categorized into two phenotypes: classic and non-classic (later-onset), each exhibiting a wide phenotypic spectrum. The more severe classic form generally begins in childhood and exhibits a more consistent and insidious pattern of disease progression [5]. Presenting signs and symptoms often include neuropathic pain, heat intolerance, and gastrointestinal issues [8, 9]. Additional signs and symptoms include the following: hypohidrosis progressing to anhidrosis, dermal angiokeratoma (a skin finding commonly found in FD) [5], proteinuria, bradycardia, and cornea verticillata (a characteristic corneal opacity highly suggestive of FD). Without initiation of FD-specific therapy, the disease may progress to chronic renal disease, left ventricular hypertrophy (LVH), arrhythmias, hearing loss, transient ischemic attacks (TIA), stroke, and ultimately premature death [5]. Individuals with the more prevalent nonclassic phenotypes have varying levels of residual α-Gal A activity and therefore experience a more variable disease course with symptoms that typically emerge after childhood and include LVH, arrhythmias, and/or markedly decreased glomerular filtration rate [5, 10]. FD occurs in people of all ages in diverse ethnic, racial, and demographic groups. Estimates of incidence vary depending on the screening methods and populations studied [11]. Initially, it was estimated that classic FD affected 1:117,000 live male births globally [12]; however, the first newborn screening studies conducted in the USA and internationally demonstrated a much higher prevalence of 1:1250 to 1:11,854 with a much higher representation by patients with nonclassic FD [9]. Numerous screening studies of high-risk populations have identified FD in patients with end-stage renal disease [13–16], cardiac hypertrophy [16–19], unexplained left ventricular hypertrophy [20, 21], and stroke [22–25]. It is significant that many screening projects utilized only α-Gal A to identify at-risk patients which reportedly can miss detection of 30–40% of females with FD [5]. In addition to high-risk population screening studies and newborn screening, artificial intelligence (AI) methods are currently being developed to screen for FD [26, 27].

Compared to the general population, without treatment, life expectancy for people with FD decreases by 20 and 10 years in males and females respectively [10]. The current therapeutic options for FD vary in availability by country but include the following: intravenous enzyme replacement therapy (ERT) with recombinant α-Gal A [agalsidase alpha (Replagal**®**, Takeda), agalsidase beta (Fabrazyme**®**, Sanofi), pegunigalsidase alfa-iwx (Elfabrio**®**, Protalix Biotherapeutics)], and the oral small molecule pharmacologic chaperone migalastat (Galafold**®**, Amicus Therapeutics) for those with amenable *GLA* variants. Several other treatments are in development including the following: second-generation ERT, moss-aGal (Greenovation Biotech/Eleva); substrate reduction therapies, venglustat (Sanofi) and lucerastat (Idorsia Pharmaceuticals); and mRNA and gene therapies, isaralgagene civaparvovec (ST-920, Sangamo) and 4D-310 (4D Molecular Therapeutics).

Like most rare diseases, there is a lack of awareness of FD. Additionally, there is great variability in clinical presentation, with many patients presenting with non-FD-specific renal and cardiovascular diseases. Without properly directed further investigation, patients often are misdiagnosed and endure long diagnostic delays [28].

To address the critical need for earlier diagnosis, we have developed a statistical risk prediction model, the FDrisk, which is a validated, easily accessible, clinician-friendly, online, risk-assessment scoring tool for predicting an individual’s risk for FD and prompting diagnostic testing and clinical evaluation.

## Methods

This study received approval from the Emory University Institutional Review Board (IRB no. 00003599). Utilizing electronic health records, data were collected retrospectively from randomly selected, deidentified patients with confirmed FD treated at the Emory Lysosomal Storage Disease Center from 2001 through 2020. As a referral center of excellence for FD, the patients are from beyond the southeast region of the USA. The patients’ original clinical evaluations were undertaken by genetic counselors and medical geneticists with an expertise in FD. The diagnosis of FD was documented by evidence of a pathogenic variant in *GLA* and/or an abnormal level of leukocyte α-Gal A. Additional evidence of FD included the presence of symptoms consistent with FD and/or a family history of FD. For patients treated with ERT, information was only gathered for the time period previous to receiving treatment.

Based on the opinion of clinical experts in FD, i.e., healthcare providers with clinical and research experience working with more than 100 patients with FD over a decade of time, 30 characteristic clinical features of FD, including relevant demographic information, were initially identified. Subsequently, these features were curated into 16 clinical covariates used as predictors for the risk of FD (Table 1).

**Table 1:** Selected characteristic clinical features of Fabry disease with corresponding curated clinical covariates

Curated clinical covariates	Selected characteristic clinical features of Fabry disease
Sex	Sex
Age at testing	Age at testing
Cornea verticillata	Cornea verticillata
Hypohidrosis	Hypohidrosis
Heat/cold intolerance	Heat/cold intolerance
Angiokeratoma	Angiokeratoma
Pain unspecified	Acute burning extremity pain, chronic burning extremity pain, joint pain
Fatigue	Fatigue
Numbness/tingling	Numbness; tingling
Tinnitus	Tinnitus
Proteinuria	Proteinuria
Renal failure	Renal failure
Cardiac disease	Arrhythmia, bradycardia, conduction abnormality, left ventricular hypertrophy, cardiac valvulopathy
Transient ischemic attack/stroke	Transient ischemic attack, stroke
Depression/anxiety	Depression, anxiety
Gastrointestinal disease	Nausea, vomiting, constipation, abdominal pain, diarrhea

Retrospective chart review was conducted to identify FD cases and controls for this study. A total of 130 diagnosed FD cases were used as the FD case cohort for training FD risk prediction models. The FD case cohort ranges in age from 0 to 76 years old (a few patients were diagnosed prenatally or at birth) and is composed of 46 males and 84 females. Another 30 diagnosed FD cases (12 males and 18 females; ages ranging from 0 to 65 years old) were reserved for testing the FD risk prediction models.

Deidentified, FD-negative controls were randomly selected from the Fabry Disease Diagnostic Testing and Education project database, a program within the American Association of Kidney Patients (AAKP) Center for Patient Education and Research. Through this program, no-cost testing is available for individuals who have health issues seen most often in FD and/or have a known family history. Individuals complete a self-response questionnaire indicating the presence or absence of 16 possible FD-related signs/symptoms. In addition, family members with a confirmed diagnosis of FD are identified.

Clinical data for participants in the AAKP program, who are listed in the database for the time period from 2007 to 2015, were retrospectively reviewed. The available database only listed people with a positive family history of FD. A total of 297 controls with complete observations of these 16 clinical features were selected. These include 130 controls (46 males and 84 females matched with the same gender ratio and sample size of the case group, ages ranging from 1 to 81 years old) for model training, and 167 controls (71 males and 96 females, ages ranging from 1 to 96 years old) for model testing.

In order to harmonize clinical features of the FD case and control groups, the more specifically described clinical features collected for the FD case cohort were grouped into the16 broader pre-established AAKP clinical categories. For example, for the AAKP clinical category “cardiac disease,” it was necessary to combine the following individual cardiac features found in the FD case group: “arrhythmia,” “bradycardia,” “conduction abnormality,” “left ventricular hypertrophy,” and “cardiac valvulopathy” (Table 1). When identical clinical variables were not available, data was collected as specifically as possible. Note that several variables were identical, e.g., “tinnitus.”

### Statistical methods

Training and testing samples were randomly selected from the whole cohort. In the training cohort, we first randomly selected 130 cases from all cases in our cohort and randomly selected the same number of controls (130) as the cases. The training cohort with 260 samples (168 females, 92 males, equal case/control ratios for females and males) was used to train the risk prediction models for FD [29]. A total of 15 clinical covariates (except the sex variable) were considered to fit the sex-specific risk prediction models, and a total of 16 clinical covariates were considered to fit the risk prediction model for all samples. A small percentage of missing values were observed in the FD cases and were imputed to the median values of FD cases of the corresponding covariate. The missing rates for FD cases were < 4% for clinical variables except proteinuria (17%) and cornea (36%).

Because all members of the control cohort had a positive family history of FD while not all members of the FD case cohort had a positive family history, including the family history as a predictive variable in model training would have led to a negative effect size, which would have suggested that a sample with a positive family history of FD has less risk than one without. To avoid such bias caused by training data curation, the covariate of family history of FD was excluded from our model training with plans to annotate the final tool with a note that all patients with a family history of FD should be considered “at risk.”

Logistic regression models with elastic-net penalty [30] were trained for FD cases vs. controls, respectively, for all samples, female samples, and male samples in the training cohort (*n* = 260). The elastic-net penalty is a weighted combination of L1 (lasso) [31] and L2 (ridge) [32] penalty with one parameter alpha denoting the proportion of L1 (lasso) penalty and another parameter lambda denoting the shared penalty magnitude. The L1 (lasso) penalty helped select predictive covariates, while the L2 (ridge) penalty helped handle correlated covariates. Both parameters were tuned by 10-fold cross validation [33] where the values leading to the highest classification accuracy by 10-fold cross-validations were used to train the risk prediction models.

A test cohort with 197 independent samples (30 cases and 167 controls, 114 females, 83 males) was used to test the model performance. Probabilities of having FD were predicted per sample by these fitted logistic models for test samples. Predicted probabilities were obtained for all test samples by using the risk prediction model fitted using all samples. Additionally, another set of predicted probabilities of female test samples was obtained by using the risk prediction model fitted using only female training samples, while another set of predicted probabilities of male test samples was obtained by using only male training samples.

The prediction performance was first evaluated by using the prediction area under curve (AUC) values of the receiver operating characteristic (ROC) curves [34]. The AUC value indicates the probability that a randomly selected true case will have higher risk probability than a randomly selected true control. A threshold of 0.5 was selected to determine a predictive “case” for those with predicted probability ≥ 0.5 and a predictive “control” for those with predicted probability < 0.5. By applying this threshold, the prediction accuracy of the overall risk prediction model and two sex-specific risk prediction models was evaluated in independent test samples using the overall prediction accuracy, sensitivity, and specificity. Test results were further stratified by age.

## Results

**Fig. 1:**
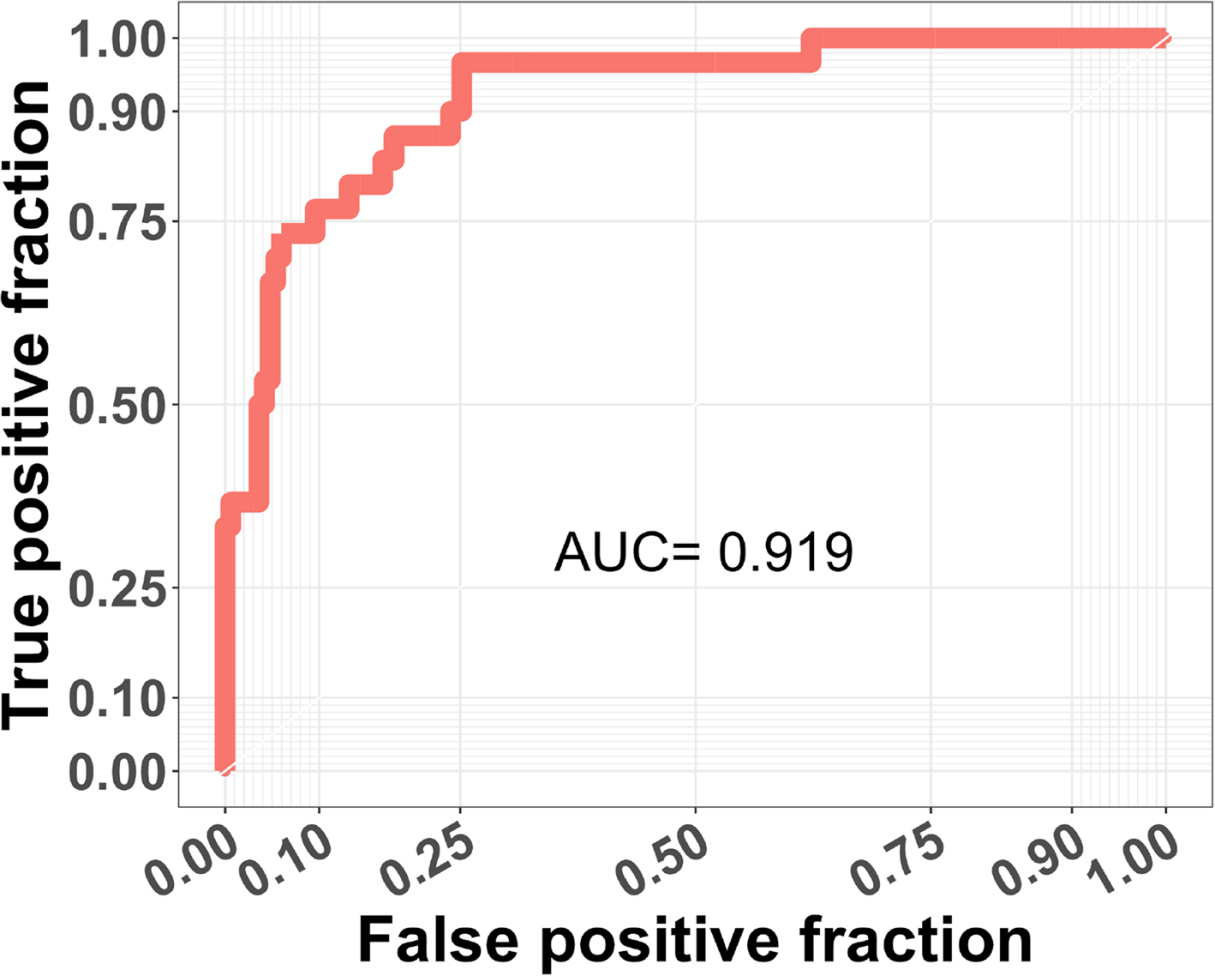
Fabry disease risk prediction model, ROC plot: risk prediction results of test samples for the full analysis set

**Fig. 2:**
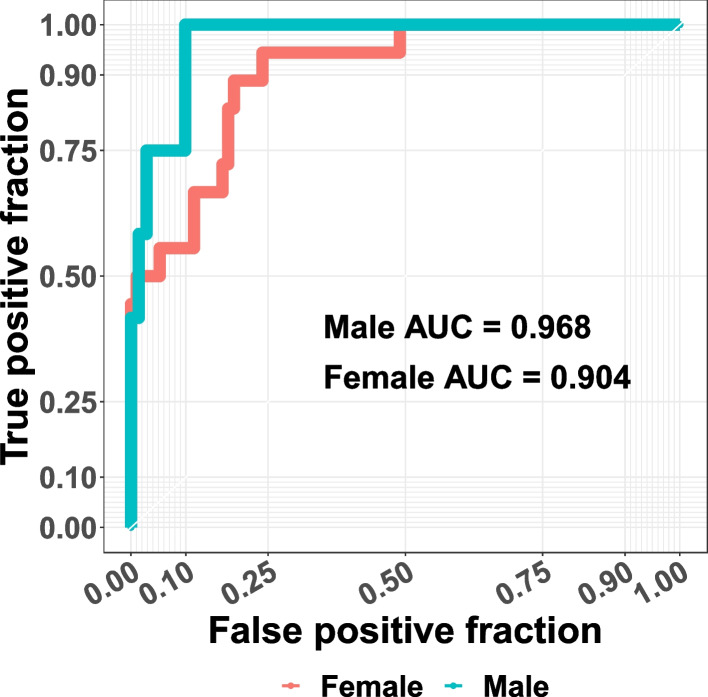
Fabry disease risk prediction model, ROC plot: risk prediction results of test samples for males and females

All models performed very well, with ROC curves and AUC values calculated to determine the performance of each model: full analysis set (FAS) 0.919 (Fig. 1), male model 0.968, and female model 0.91 (Fig. 2).

Here, although high AUC values were partly due to a large proportion of controls in the test cohort, the prediction model with test AUC > 0.9 is still considered to be very accurate. Higher AUC values, generally ranging between 0.5 and 1, indicate higher prediction accuracy by the corresponding risk prediction model.

**Fig. 3:**
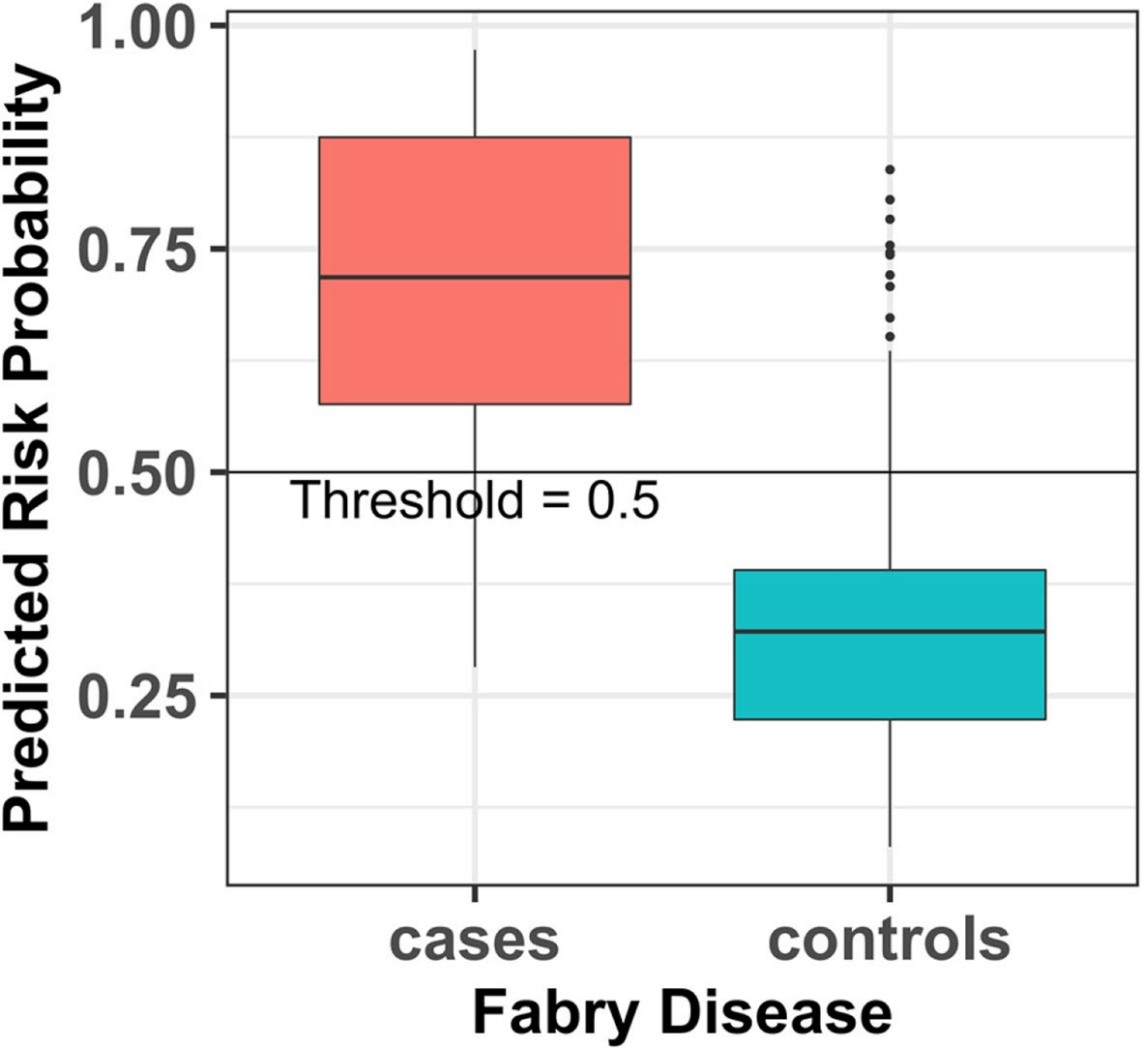
Fabry disease (FD) risk prediction model, box plot: predicted risk probabilities of true FD for test samples for the full analysis set

**Fig. 4:**
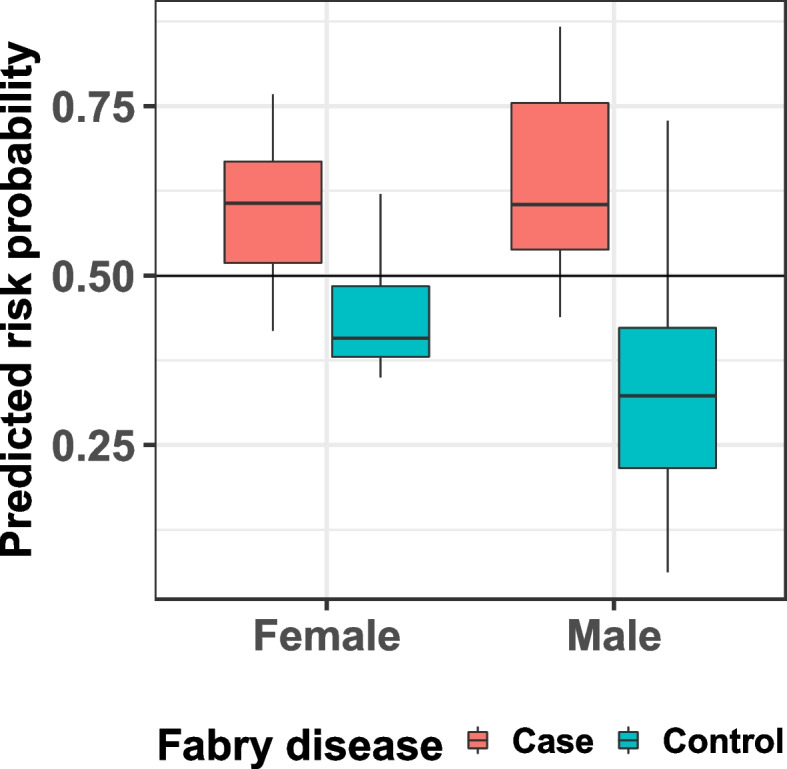
Fabry disease (FD) risk prediction model, box plot: results of predicted risk probabilities of true FD for test samples for males and females

A risk prediction threshold of ≥ 0.50, which was chosen to determine if a patient is at risk for FD, was shown to provide the best balance between sensitivity, specificity, and positive predictive value in the test results (Fig. 3 and Fig. 4).

That is, a risk score of 50% or greater is categorized as “at risk” for FD. In the test cohort, the overall risk prediction model demonstrated 80% sensitivity, 83.8% specificity, and a positive predictive value of 47.1%; the male-specific risk prediction model demonstrated 75% sensitivity, 95.8% specificity, and a positive predictive value of 75%; and the female-specific risk predication model demonstrated 83.3% sensitivity, 81.3% specificity, and positive predictive value of 45.5%. Stratification of the test cohort by age ≥ 18 years old (*n* = 136) or < 18 years old (*n* = 61) with the FAS model showed a greater AUC of 0.964, a greater specificity of 94.1%, and a greater accuracy of 91.8% in the < 18-year-old age group compared with an AUC of 0.912, specificity of 79.3%, and accuracy of 79.4% in the ≥ 18-year-old age group. Comparable sensitivity of 80% was shown for both age groups. The finding of greater AUC, specificity, and accuracy in the < 18-year-old age group is consistent with higher likelihood that pediatric patients with the characteristic features of FD have the disease.

**Fig. 5:**
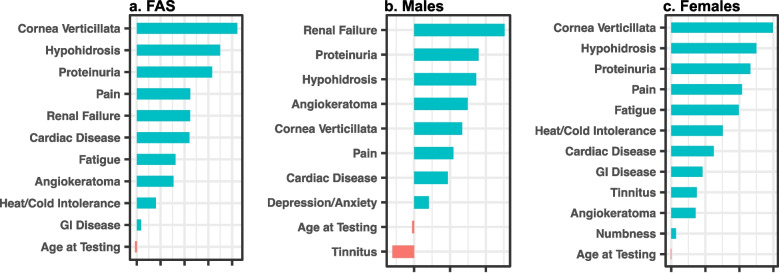
Effect size. Estimated coefficients (beta) of predictive clinical variables in Fabry disease risk prediction models. **a** Full analysis set (FAS). **b** Males. **c** Females

The estimated coefficients of predictive clinical variables were determined for the overall FAS model, as well as for the male- and female-specific models (Fig. 5).

The magnitude of the coefficients reflects the magnitude of influence of the predicted risk probability by the corresponding clinical variables, i.e., a variable’s effect size. Positive coefficients mean that the present or larger values of the corresponding clinical variables will increase the risk probability, while negative coefficients indicate that the present or larger values of the corresponding clinical variables will decrease the risk probability. All clinical variables with non-zero coefficient values would contribute to the predicted risk probability. A clinical variable with a high positive coefficient, such as cornea verticillata, reflects the disease-specific manifestations of FD. The clinical variables with coefficients of smaller magnitudes are not necessarily significantly associated with FD but would be more concerning when seen at an earlier age or in combination with features that suggest a diagnosis.

## Discussion

Clinicians in both general practice and many nongenetic focused sub-specialties are likely to be caring for people with undiagnosed FD. However, lack of awareness of FD, difficulty recognizing the clustering of pathologies, vague subjective complaints, and the lack of a detailed family history can cause long diagnostic delays for patients with FD and their affected family members. Cascade testing after diagnosing a patient with FD is critical since, on average, each patient with FD has at least five family members who also have FD [35]. Additionally, it needs to be recognized that females can be severely affected by FD, and that the number of females with FD has been profoundly underestimated partly due to limited testing of only α-Gal A without follow-up molecular testing of the *GLA* gene [5].

**Fig. 6:**
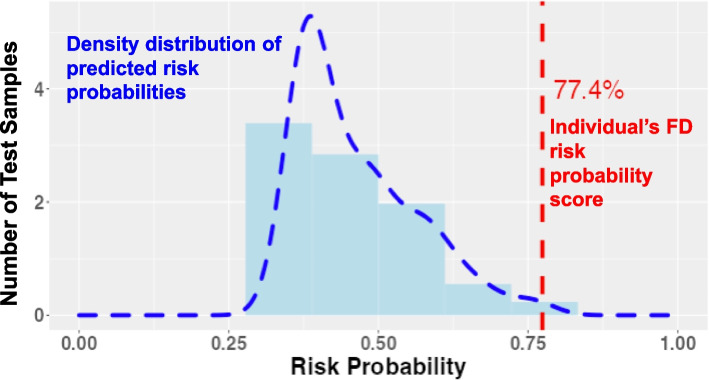
FDrisk risk score generation. Online, real-time, interactive graphic display of the predicted Fabry disease risk probability distribution

With availability of effective treatment and support options, increasing understanding of FD and the significant impact on quality of life [36], it has become even more imperative that people with FD are identified earlier. To address this need, we developed a statistical risk-prediction model, the FDrisk, which is a validated, easily accessible, and clinician-friendly, online, risk-assessment scoring tool for predicting an individual’s risk for FD and prompting diagnostic testing and clinical evaluation (Fig. 6).

As a result of a collaborative effort, the FDrisk tool can be accessed at FDrisk.org which is hosted and maintained by the ThinkGenetic Foundation, Inc. (thinkgenetic.org), a nonprofit organization dedicated to creating practical, informational content on genetic diseases, and useful resources. On the web page, the FDrisk appears as a real-time graphic display of the risk score which is generated by answering 16 clinical questions requiring a yes/no response. The risk score is clearly expressed as a percentage of predicted risk probability and is marked on the graph with a vertical red line. Anyone generating a risk score of 50% or greater should be sent for clinical evaluation and diagnostic testing, which is available through sponsored testing programs and standard laboratories. Although the most important risk factor of family history was unable to be included in the analysis, a notation on the tool reminds clinicians that anyone with a family history of FD is considered to be at high risk for FD, no matter the risk score, and should be sent for diagnostic testing.

In addition to the FDrisk, screening tools using AI methodology are currently in development and have great future potential to identify undiagnosed patients with FD. One such tool, the OM1 Patient Finder™ (OM1 Inc., Boston, MA, USA), uses deidentified longitudinal health history data and predictive analytics to identify patients most likely to qualify for, and participate in, specific trials [37]. This includes patients who may have relevant but undiagnosed disease. Using data, e.g., medication history, prescription information, laboratory results, symptoms and signs, procedures, and diagnoses extracted from patient-level health care claims and electronic medical records Jefferies et al. [26], examined the performance of the OM1 Patient Finder in identifying patients with FD by looking at the phenotypic patterns of a study population of 1,004,978 patients which included 4978 patients with confirmed FD. They concluded that the tool has “a very strong analytic performance” in identifying patients with FD, and that it may contribute to increasing the diagnosis of FD.

Michalski et al. [27] developed a decision-support scoring system for FD, using natural language processing (NLP), to evaluate the electronic health records of 19,385 patients, including 13 with FD from a multi-hospital Polish healthcare system. Based on physician opinion, 13 curated clinical features of FD were chosen and scored. The FD risk score was determined by the sum of the scores for each clinical feature. A patient with a risk score of ≥ 4 was further evaluated. One patient in the control group with a score of eight as well as one patient from the study group with a score of three were ultimately diagnosed with FD. Although the scoring system achieved an AUC of 0.998, there were several limitations cited by the authors which impacted the accuracy and discrimination power in identifying patients with FD. The authors concluded that further development and testing with a larger and more diverse patient population are needed.

When studying rare diseases, it is often a challenge to curate cohorts with hundreds of samples. However, in this retrospective chart review, a relatively large number of patients with confirmed FD and relevant clinical data were identified [38]. Nonetheless. there are a number of limitations given our available data. First, in order to match clinical features for the analysis, several detailed individual features in the case cohort were grouped to fit into some of the more broadly defined pre-established categories of clinical features in the AAKP control cohort.

Second, due to limitations in the AAKP database, all the patients in the control cohort had a positive family history of FD. Since not all patients in the case cohort had positive family history of FD, family history was excluded in the final analysis to avoid bias caused by training data curation. Including family history would have falsely indicated that those with a positive family history have less risk of having FD. Nonetheless, as instructed in the tool, anyone with a family history of FD needs to be tested no matter the resulting risk score. It is important to note that all three prediction models (full analysis set, male and female) performed very well as determined using ROC curves and AUC values. The resulting AUC values, greater than 0.9 for all three models, indicate a high prediction accuracy by these risk prediction models.

The third limitation, ascertainment bias, is present since both the case and control cohorts were collected from single centers.

Future directions are to pilot the FDrisk in clinics such as cardiology, nephrology, and neurology to determine its clinical efficacy and promote awareness of both FD and this tool. Depending on these results, a “next-generation” tool may be generated utilizing an expanded training data set which would include case and control databases with additional equally matched clinical variables, including family history.

At this time, the FDrisk, as an open and easily accessible, user-friendly tool, can generate a risk score in the clinic using information directly obtained from the patient. With its ease of use, and availability online, investigators may choose to study the clinical efficacy of the FDrisk in their own populations*.* Eventually, the FDrisk tool could be used in the screening of a large number of patients by applying it to data captured from the electronic medical records of selected groups in concert with an AI-based model.

## Conclusion

We believe that the implementation of this FDrisk tool will have a positive impact on people with FD and their families by increasing the number of people screened for FD, decreasing the frequency of missed and misdiagnoses, and leading to earlier and correct diagnosis. Once identified, patients can be appropriately treated by clinicians experienced in the care of individuals with FD along with a team of multidisciplinary specialists who can provide appropriate drug treatment, organ-specific management, treatment of variable adjuvant conditions, genetic counseling, and psychosocial support. Initiating earlier disease-specific therapy could slow disease progression and development of complications [39, 40] and ultimately make a meaningful difference in the lives of people living with FD.

## Data Availability

The data sets analyzed during the current study are not publicly available because they are confidential patient records, protected by the Health Insurance Portability and Accountability Act, but the data may be available from the corresponding author on reasonable request.
